# Pseudorandomised controlled trial of a novel navel barrier solution versus 10% iodine to protect navel and ear tag sites of neonatal lambs

**DOI:** 10.1002/vetr.70169

**Published:** 2025-12-26

**Authors:** Fiona M. Lovatt, Jonathan J. Powell, Carlos A. P. Bastos, Ian S. McCrone

**Affiliations:** ^1^ Flock Health Ltd Barnard Castle UK; ^2^ University of Nottingham Nottingham UK; ^3^ University of Cambridge Cambridge UK; ^4^ NoBACZ Healthcare Cambridge UK

**Keywords:** clinical trial, ear tagging, lamb mortality, navel treatment, neonatal disease

## Abstract

**Background:**

During lambing, 10% iodine is often used to protect neonatal navel and ear tag sites. The evidence for its effectiveness is sparse. Recently, a specific navel barrier solution (NBS) was developed. Here, an in vivo field trial compared the two treatments.

**Methods:**

Ten farms reported data from 6840 lambs. The navel and ear tag sites of alternate lambs were treated with usual care‐iodine or NBS. Usual care‐iodine was 10% iodine for all navels and for most ear tag sites.

**Results:**

Mortality was 8.36% for the usual care‐iodine group and 9.34% for the sub‐group where only iodine was used for navel and ear tag sites. It was 6.51% for NBS (*p* < 0.004 vs. iodine groups). Mixed‐effect generalised linear modelling gave an adjusted odds ratio (OR) for mortality of 0.76 for NBS (95% confidence interval [CI]: 0.63–0.91; *p* = 0.003). Spraying navels was inferior to dipping them for survival (OR 2.14; 95% CI: 1.11–4.09; *p* = 0.02). Farmers did not report differences in joint ill between the groups.

**Limitations:**

For practical implementation, the study was non‐blinded and pseudorandomised. Non‐appearance of a lamb at the week 8 weighing event was taken as a sensitive and objective proxy for death.

**Conclusion:**

Optimal navel and ear tag site application during lambing efficiently protects against neonatal deaths. NBS is superior to 10% iodine, and dipping outperforms spraying for improvement of lamb survival.

## INTRODUCTION

The umbilical cord, as the blood supply between the fetus and placenta during pregnancy, ruptures during the last stages of the birthing process. This severs the umbilical arteries and vein and leaves a remnant of cord attached to the lamb abdomen. If unprotected, this stump can be a site of pathogen entry resulting in a localised umbilical infection (navel ill), a localised or diffuse serofibrinous and/or suppurative peritonitis, abscessation of the liver and other organs, or septicaemia that may develop into infectious arthritis (commonly termed joint ill).^1^, ^2^, ^3^ In the UK, ovine *Streptococcus dysgalactiae* subspecies *dysgalactiae* is the most frequent cause of joint ill in newborn lambs, with a previously reported prevalence of up to 50% of lambs and a subsequent mortality of 20% of cases.^4^, ^5^ Outbreaks of joint ill^6^, ^7^, ^8^ have been shown to be significantly associated with environmental and flock‐level risk factors rather than associated with either lamb or ewe factors, although infection from ear tagging may also play a role.^7^, ^8^, ^9^

Navel ill and ear tag infections, and thus the potential for downstream joint ill, are managed prophylactically in newborn lambs. Some producers still administer prophylactic antibiotics in the first few days of life^5^, ^10^ despite this practice being considered inappropriate.^11^ Instead, typically, a 10% iodine solution (iodine in alcohol/water) is applied to the umbilical area straight after birth. Dipping of the umbilical stump and surrounding area is thought to be more effective than spraying because thorough coverage is more easily achieved.^12^ It is also sometimes recommended that dipping is repeated after 4 hours,^2^, ^3^ but there is no evidence for the effectiveness of this practice.^10^

Interestingly, despite 10% iodine being the navel treatment of choice for at least the past 20 years,^13^ there is very little evidence for its effectiveness as a preventative measure for either joint ill or septicaemia. To the best of the authors' knowledge, there have been very few such studies of lambs and none of scale in any species. In total, over the last 70 years, there have been 24 clinical trials that considered umbilical health of calves, but only four of these (17%) had over 300 animals enrolled in the trial.^14^ Three studies that directly compared umbilical treatments reported no difference in umbilical cord healing rates, external umbilical infections or subsequent systemic disease following the use of either iodine or chlorohexidine gluconate compared with chlorine, trisodium citrate,^15^ nisin^16^ or a commercial solution.^17^

There have been question marks over the longevity of the iodine market from several standpoints, namely, potential for use in the illicit drugs trade,^18^ endocrine disruption^19^ and supply issues, which has led to a variety of prophylactic navel treatments being proposed.^20^ It is generally agreed that the key to any success is rapid desiccation (‘drying’) of the umbilicus while rendering the applied area clear of bacterial colonisation that could, otherwise, ingress systemically.^21^ In this respect, a specific navel barrier solution (NBS), comprising the biopolymer shellac plus low levels of zinc and iron ions that are dissolved in ethanol, has recently been developed.

This randomised controlled trial was therefore set up to formally compare the effectiveness of NBS versus 10% iodine for navel application in lambs. Data from commercial sheep flocks around lambing time are notoriously difficult to obtain.^22^ For these reasons, while the study was designed using REFLECT reporting guidelines,^23^ it was pragmatically limited to making minimal changes to usual flock procedures and recording.

## MATERIALS AND METHODS

### Trial design

In keeping with the above, the trial was designed for lambing personnel to do nothing out of their usual practice other than alternating between usual care (lambs given odd‐numbered ear tags) and NBS (lambs given even‐numbered ear tags). They used their standard methods of application for both treatments, and recorded data according to their usual practice. Blinding was not possible because the two treatments had different colours. The study was approved by the Ethics and Welfare Committee, Department of Veterinary Medicine, University of Cambridge (CR781).

The primary outcomes were on‐farm‐reported joint ill and mortality, and the secondary outcome was follow‐up weights. Clinical diseases (joint ill, navel ill and ear tag infections) were variously recorded by shepherds in each flock, with the list of lamb numbers supplied at the end of lambing. The trial ran from December 2023 to April 2024, and an upper limit for recruitment numbers was not set but the lower limit was 1536 total lambs. The sample size was calculated for tests of equivalence or superiority. The power calculation assumed a significance of *p*‐value of 0.05 (alpha value) and a power of 80% (beta value) and set the incidence of joint ill for iodine‐treated animals as 1%. As such, an incidence of 3% or more in the NBS group would show inferiority, whereas 0% would show superiority.

For welfare reasons, it was a condition of the ethical approval that joint ill and mortality were monitored in real time. The trial would need to have stopped with immediate effect if, any time from 12 events onwards in the NBS group, the incidence was greater than that in the iodine group by an absolute 3% or more. All participants were encouraged to take diseased or dead lambs for veterinary examination, although in practice, this was very rarely undertaken.

### Recruitment and data recording

Ten farms with 11 flocks were recruited across England and Wales by the lead author (F.M.L.) and are summarised in Table 1. Farms were professionally managed and traditionally treated at neonatal navel sites with 10% iodine solution and were intended to meet the following criteria: ear‐tagging lambs with unique identification within 48 hours of birth; electronic recording of lamb birth weights (by hanging from a suspended scale) and mortality; and recording lamb weights at approximately 56 days of age (follow‐up weights) and they were prepared to treat ear tag sites. Lamb breeds included both purebreds and crosses of the following breeds: Charollais, Berrichon, Mule, Meatlinc, Texel, Suffolk, Hampshire, Composite, Beltex, Lleyn, Teeswater, Aberfield, Highlander, Welsh, Oxford Down, Suftex, Zwartble, Belclair, Llanwenog, Kerry Hill, Treform, Chuff, Romney and Charmoise.

**TABLE 1:** Description of flocks in the trial with approximate locations, lambing dates and numbers of lambs born and numbers that died.

Farm	Location	Lambing period	Navel treatment	Control ear tag treatment	Sex of lambs			Total	Died	Mortality (%)
					Female	Male	Castrated male			
A	Leicestershire	1/12/23–2/1/24	Dipped	Iodine	86	77	0	163	6	3.68%
B	North Yorks	1/12/23–11/12/23	Sprayed	Antibiotic spray	183	185	0	368	12	3.26%
C	Hampshire	29/12/23–25/1/24	Dipped	Iodine	51	64	0	115	6	5.22%
D	Cheshire	6/1/24–13/1/24	Sprayed	Iodine	51	48	0	99	22	22.22%
E	Cornwall	26/2/24–24/4/24	Sprayed	Iodine	370	1	446	817	63	7.71%
F	Powys	4/3/24–4/4/24	Sprayed	Iodine	70	81	4	155	15	9.68%
G	North Yorks	20/2/24–12/5/24	Sprayed	Iodine	259	173	50	482	48	9.96%
H	Powys	3/3/24–21/5/24	Sprayed	Iodine	493	1	496	990	116	11.72%
I	North Yorks	17/3/24–21/4/24	Dipped	Various^a^	412	24	391	827	57	6.89%
J	Leicestershire	18/3/24–2/6/24	Sprayed + dipped	Iodine	1145	0	1086	2231	140	6.28%
K	Carmarthenshire	7/4/24–4/5/24	Dipped	NBS	286	212	93	593^b^	23	3.88%

*Note*: Flocks B and G were two different lambing periods for the same farm.

^a^ See text for details of ear tag treatments for flock I.

^b^ Two lambs with unstated sex.

Data collection relied on usual flock procedures, involving hand‐held recording devices that uploaded data into commercial software packages (FarmIT; Border Software for seven flocks and FarmWorks; Shearwell Data for four flocks) that could export a comma‐separated values text file into Microsoft Excel. These outputs were then emailed to the lead author.

Some flocks (A, F, H, I and K) had different personnel tagging with multiple flock numbers simultaneously, so it was not always possible to apply an odd tag for iodine lambs and an even tag for NBS lambs. In these flocks, each person alternately allocated iodine or NBS immediately following birth at the time of navel treatment, and treatment type was added as a ‘lamb trait’ on the hand‐held device at ear tagging, which was the time at which other traits (e.g., sex, birth weight) were recorded. As such, there was inexact matching of numbers between the groups, with 49.5% in the iodine group and 50.5% in the NBS group.

### Treatments and outcomes

All iodine products were described on the labels as 10% British Pharmacopoeia (BP) strong iodine solution. Flock D used ‘Triamvet’ (iodine, potassium iodide, N‐(3‐aminopropyl)‐N‐dodectylpropane‐1,3‐olamine in spirits to BP Standards). Flocks H and J used ‘Nettex’, and flock E used ‘Mole Valley Farmers’, with an additional 40 mL spirit in each litre. For flock C, generic ‘10% BP strong iodine solution’ was reported. For the remaining flocks, ‘Battles’ iodine was used. NBS was from NoBACZ Healthcare (NoBACZ Navel; also termed Umbirez). Any additional animal husbandry was as per normal flock practice. Only live lambs had navel treatments applied and all were included in the study outcomes.

Each farm reported its method of navel application (i.e., dipping or spraying, and single or repeated) and followed exactly the same protocol for both groups. Flock J sprayed all navels at birth and then dipped them approximately 12 hours later.

When it came to ear tag sites, all lambs of the NBS navel group also had NBS used for the ear tag sites. Similarly, all lambs of the iodine‐navel group also had iodine used for their ear tag sites except for flocks B, I and K as follows: flock B ear tag sites (of iodine‐navel lambs) received oxytetracycline spray, and flock K ear tag sites (of iodine‐navel lambs) received NBS. Flock I ear tag sites (of iodine‐navel lambs) were untreated from 17 to 29 March and then treated with surgical spirit from 30 March to 5 April and then with NBS from 6 April until the end of lambing on 21 April.

Mortality records and the time of death were not accurately maintained, so absence at the follow‐up weighing event was used as a sensitive and objective proxy for death in these flocks that routinely weighed and recorded all lambs at around 8‒10 weeks of age for accurate breeding records. There were no significant adverse events (such as unexpectantly high numbers of lambs with the same disease or infection) indicated in the flock treatment records or the very limited number of postmortem examinations undertaken.

The chi‐square test was first used to compare all‐cause mortality between the groups. Then, to guard against possible confounding factors, a mixed‐effect generalised linear model with a binomial error term was also used to evaluate the effect of navel treatment on pre‐weaning all‐cause mortality. The following categorical variables were included as fixed effects: type of navel treatment (NBS vs. iodine), sex (female, entire male and castrated male) and method of application (dipped only, sprayed and then dipped, or sprayed only). A random intercept for farm was included to account for clustering of lambs within farms. Interaction terms between treatment, sex and application method were tested using likelihood ratio tests. Non‐significant interaction terms were dropped from the final model. A confirmatory meta‐analysis, treating each flock as a separate study, was also performed (Figure S1). All the statistical analyses were conducted using Stata version 11 (StataCorp).

For the secondary outcomes, first, weights adjusted to day 56 were used and were calculated as: [birth weight + (56 × daily weight gain) in kg] for each lamb. The daily weight gain itself was calculated as: follow‐up lamb weight (kg) minus birth weight (kg), divided by lamb age (days). Only flocks that recorded both a birth weight and a follow‐up weight could be included in the analysis (*n* = 3368 lambs, which were from all flocks except for flocks C, J and K, as only a common estimated birth weight [4 kg] was recorded for these). Animals with extremely low daily weight gain (<0.05 kg/day) or birth weights above 15 kg were treated as likely data collection errors, and therefore, excluded from data analysis. Second, the follow‐up weights of twins, which inevitably had one treated with iodine and one with NBS, and for which the twin pairs were measured within 24 hours of each other, were also compared.

Deaths associated with on‐farm‐diagnosed joint ill were very low overall and were compared between the groups by Fisher's exact test. The secondary outcomes are presented as averages with associated 95% confidence intervals (CIs).

## RESULTS

Nine farms with one lambing period and one farm with two lambing periods were entered into the study with the demographic data shown in Table 1. Each farm recruited between 99 and 2231 lambs during their lambing periods.

During the study period, the farmer‐reported incidence of joint ill was 2.7%, with 93 cases in each navel treatment group: of these, 22 (23.7% of those reported) died in the iodine‐treated group, and 13 (14.0%) died in the group treated with NBS (*p* = 0.09; Fisher's exact test). The all‐cause mortality of lambs in the study, however, was considerably higher with 508 of the 6840 recruited lambs (7.4%) recorded as dying during the study period.

**FIGURE 1:**
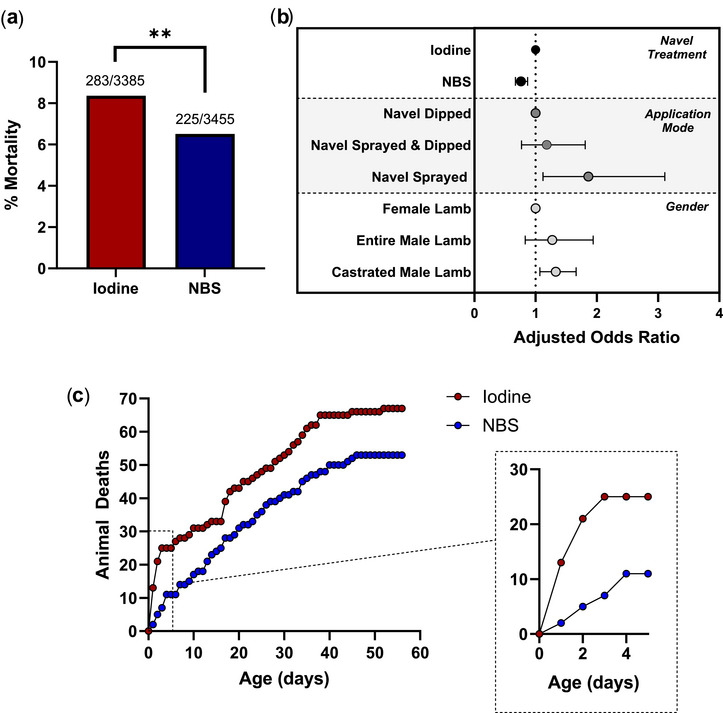
All‐cause mortality data. (a) Percentage mortality for all lambs: ‘NBS’ is the navel barrier solution group, and ‘iodine’ is the usual care‐iodine group^a^ (^**^ *p* < 0.004 for NBS vs. iodine by chi‐square test). (b) Forest plot showing adjusted odd ratios for different sub‐groups, based on navel treatment, type of treatment application and lamb sex. Numbers are shown in Table 3. (c) Cumulative deaths over a period of 8 weeks and, inset, the first 5 days of that 8‐week period magnified. ^a^For the sub‐group of 2505 lambs that had iodine applied to both navel and ear tag sites, mortality was 9.34% (*p* < 0.004 vs. NBS by chi‐square test).

In the usual care‐iodine group, of 3385 lambs, 283 died (8.36%). Of these, 880 lambs received a variety of non‐iodine ear tag treatments, including surgical spirit, antibiotics and NBS (see the ‘Methods’ section). However, for the 2505 lamb sub‐group that had iodine applied to both navel and ear tag sites, the mortality was further increased to 9.34%. These figures were compared to a mortality of 225 out of 3455 lambs (6.51%) for those treated with NBS (*p* < 0.004 vs. usual care‐iodine or the sub‐group by chi‐square test; Figure 1a).

For the data modelling a total of 6838 lamb observations from 11 flocks were included; two lambs were excluded from the dataset as they had missing data for sex. A breakdown of the data across all variables is shown in Table 2 with the univariate analyses.

**TABLE 2:** Summary of lamb deaths by treatment, sex and treatment type with univariate analysis showing odds ratios (ORs) and 95% confidence intervals (CIs) plus associated *p*‐values.

Variable	Category	Number of lambs	Number died	Percent died	OR	95% CI	*p*‐Value
Overall	‒	6840	508	7.43%			
Treatment	Iodine	3385	283	8.36%	Baseline		
	NBS	3455	225	6.51%	0.76	0.64‒0.91	0.0036
Sex^a^	Female	3406	220	6.46%	Baseline		
	Entire male	866	72	8.31%	1.31	1.0‒1.73	0.0541
	Castrated male	2566	214	8.34%	1.32	1.08‒1.60	0.0057
Treatment type	Dipped only	1698	92	5.42%	Baseline		
	Sprayed and dipped	2231	140	6.28%	1.17	0.89‒1.53	0.2593
	Sprayed only	2911	276	9.48%	1.83	1.43‒2.33	<0.0001

^a^ Two animals in the iodine group were missing data for sex, so the total number in the model is 6838.

In the final model (Table 3 and Figure 1b), navel treatment with NBS was associated with a significantly lower odds of pre‐weaning all‐cause mortality compared to iodine (OR = 0.76; 95% CI: 0.63–0.91; *p* = 0.003). Entire male lambs (OR = 1.60; 95% CI: 1.14–2.25; *p* = 0.007) and castrated male lambs (OR = 1.26; 95% CI: 1.03–1.54; *p* = 0.028) had significantly higher odds of mortality than females. The application method was also associated with mortality, as lambs with navels that were treated by spraying had over twice the odds of death compared to the reference group (OR = 2.14; 95% CI: 1.11–4.09; *p* = 0.022). There was no significant effect on those lambs that were sprayed and then dipped (OR = 1.49; 95% CI: 0.53–4.19; *p* = 0.455).

**TABLE 3:** Random‐effects logistic regression model for predictors of lamb mortality (*n* = 6838 lambs from 11 flocks).

Variable	OR	95% CI	Standard error	*p*‐Value
Navel treatment				
NBS (vs. iodine)	0.76	0.63–0.91	0.071	0.003
Sex				
Entire male (vs. female)	1.60	1.14–2.25	0.279	0.007
Castrated male (vs. female)	1.26	1.03–1.54	0.131	0.028
Application method				
Sprayed and then dipped (vs. dipped only)	1.49	0.53–4.19	0.786	0.455
Sprayed only (vs. dipped only)	2.14	1.11–4.09	0.708	0.022
Constant	0.046	0.026–0.079	0.013	<0.001

*Note*: *σ*_t_ *u* (farm‐level SD): 0.443 (95% confidence interval [CI]: 0.253–0.775). *ρ* (intra‐class correlation): 0.056 (95% CI: 0.019–0.154). Model fit: log likelihood = –1766.88, Wald *χ* ^2^(5) = 24.27, *p* < 0.001.

Abbreviations: CI, confidence interval; NBS, navel barrier solution; OR, odds ratio.

The intra‐class correlation coefficient was 0.056, indicating that approximately 5.6% of the total variance in lamb mortality was attributable to differences between farms/flocks. A likelihood‐ratio test comparing the random‐effects model to a standard logistic regression model showed that inclusion of farm‐level random effects significantly improved model fit (model fit: log likelihood = –1766.88, Wald *χ* ^2^(5) = 24.27, *p* < 0.001).

The generalised linear model analysis showed effects of treatment, sex and application method on lamb mortality when accounting for farm clustering. However, interaction terms between navel treatment and sex (*p* = 0.54), between navel treatment and application method (*p* = 0.86) and between sex and application method (*p* = 0.20) indicated no statistical support for interactions between these terms.

While the outcome from the random‐effects logistic regression model (Table 3) supported the direct comparison of differences in lamb mortality between the iodine and NBS groups (chi‐square test; Figure 1a) post hoc analyses for further validity were undertaken. First, accurate mortality records (i.e., with recorded dates of death) were compared between the two groups: overall, there were 120 dated records of lamb mortality at the 56‐day time point (Figure 1c). Of these, 67 were in the iodine group (55.8%) and 53 were in the NBS group (44.2%). Out to 121 days, which covered all date‐recorded deaths, there were 73 in the iodine group (57%) and 55 in the NBS group (43%). These show very similar percentages to the total recorded deaths (Figure 1a) at 55.7% for the iodine group versus 44.3% for the NBS group (Table S1), validating ‘missing‐lamb‐at‐second‐weighing’ as a suitable proxy for death. Then, an independent analysis was undertaken to confirm the outputs of the random‐effects logistic regression model. The 11 separate flocks were treated as individual studies (per flock outcomes and univariate analyses shown in Table S2), and a meta‐analysis was performed (Figure S1): the summarised hazard rate was 0.77 with a 95% CI of 0.64‒0.92 and, therefore, entirely consistent with the original model above.

Finally on mortality, it was noteworthy that the separation between the two groups was particularly marked in the first 4 days of life, after which they were approximately tracked (Figure 1c and the associated inset).

The secondary outcome was lamb weight. For 3368 lambs, weight measurements were taken at birth and, again, at some stage around 8 weeks of age. Of these, 1728 were treated with NBS and 1640 were treated with iodine. The two groups did not differ in weight at birth and, using the equation above (see the ‘Methods’ section), the mean (95% CI) adjusted weights at 56 days were 20.8 (20.6‒21.0) kg for iodine and 21.0 (20.8‒21.3) kg for NBS with overlapping CIs (Table 4). However, among this cohort, there were also 1018 twins in which each twin pair had their second weighing within 24 hours of each other. Again, birth weight averages did not differ between the two groups, and although, on average, second weights differed by 0.5 kg in favour of NBS, CIs still overlapped (Table 5).

**TABLE 4:** Analysis of 3368 lambs (1728 treated with navel barrier solution [NBS] and 1640 treated with iodine) that were weighed at birth and then approximately 9 weeks later.

	Iodine (*n* = 1640)	NBS (*n* = 1728)
Birth weight (kg), mean (95% CI)	4.6 (4.6–4.7)	4.7 (4.6–4.7)
Age at second weight (days), mean (95% CI)	66.3 (65.5–67.1)	66.5 (65.7–67.3)
Second weight (kg), mean (95% CI)	23.6 (23.3–23.9)	23.9 (23.6–24.2)
Estimated weight on day 56 (kg), mean (95% CI)	20.8 (20.6–21.0)	21.0 (20.8–21.3)

*Note*: Estimated 56‐day weights were calculated based on their daily weight gain.

Abbreviation: CI, confidence interval.

**TABLE 5:** Birth and second weights of twin pairs.

	Iodine	NBS
Birth weight, mean (95% CI)	4.6 (4.5–4.7), *n* = 509	4.6 (4.5–4.7), *n* = 509
Second weight (age 59 ± 10 days), mean ± SD	21.4 (20.9–21.9), *n* = 509	21.9 (21.4–22.4), *n* = 509
Male, mean (95% CI)	27.1 (25.1–29.1), *n* = 50/509 (9.8%)	29.3 (27.7–30.9), *n* = 50/509 (9.8%)
Castrated, mean (95% CI)	20.8 (20.2–21.3), *n* = 206/509 (40.5%)	21.2 (20.6–21.9), *n* = 215/509 (42.2%)
Female, mean (95% CI)	20.8 (20.1–21.5), *n* = 253/509 (49.7%)	20.9 (20.3–21.6), *n* = 244/509 (47.9%)

Abbreviations: CI, confidence interval; NBS, navel barrier solution.

## DISCUSSION

This study's design was intentionally uncomplicated and allowed two important factors to be prioritised in a ‘real‐life’ setting, namely: (i) recruitment of large numbers to enable a well‐powered study and (ii) alternating lambs between the two treatments to control for the many variables that may shape overall outcomes (environment, weather, husbandry, etc.).

Notwithstanding, there are limitations with a study of this type. First, true randomisation is not practical, so only pseudorandomisation can be achieved (i.e., through alternating treatments). Second, since the colours of the two treatments differ (NBS is blue and iodine is brown) operator blinding is not possible. However, through the use of 11 different commercial flocks, multiple lambing personnel and an uncomplicated study design we sought to minimise systematic observer or reporter bias, and this is supported by the data. For example, looking at the twin pair data, which provide for near‐perfect matching: (i) entire males were exactly the same in number between the NBS and iodine groups; (ii) there was a difference between the two groups for castrated males (+9 in NBS vs. iodine) and females (+9 in iodine vs. NBS) but this would not affect weights because, in the reference group (iodine) at second weighing, castrated males and females weighed the same; (iii) the above observation would have an impact on mortality, but this would have been in favour of the usual care‐iodine because female survival was significantly greater than for castrated males; and (iv) weights at birth were identical between the groups. As such, there is no objective suggestion that the ‘better lamb’ was being subliminally selected for either group.

Third, joint ill was probably not faithfully reported. The farm staff recorded their impression of clinical disease according to their normal practice which likely varied both qualitatively and quantitatively between farms. Moreover, there was no veterinary validation of farmer‐reported observations. However, joint ill was reported in identically low numbers between the groups suggesting no obvious bias.

Fourth, despite the farms of this trial being carefully and professionally managed, it is well recognised that mortality during the lambing period is, generally, not accurately recorded^22^; therefore, a proxy assessment was used, namely, lamb absence at the second weighing. For this a live lamb may have been missed inadvertently—for example, escape from the handling area or due to operator/equipment error. Notwithstanding, this was still considered the most sensitive and objective measure of mortality because erroneously missing lambs would be rare given that the second weighing event is a standard routine for flocks that rely on accurately recorded performance data to make flock breeding decisions and to sell rams and ewes at a premium. Moreover, there is no credible reason for such errors of record to favour one group above the other, especially given the 2‐month time lag between navel application and 56‐day weighing event. In support of this, the subset of total accurate, date‐recorded deaths by day 56 was 23.6% of the estimated mortality records for all lambs and showed a difference of 14 dead lambs (Table S1), which, scaled up to 100%, would be a 59 lamb difference. So the reported estimated difference of 58 dead lambs at the second weighing event (Figure 1a) is entirely consistent with the accurate, date‐recorded data (Table S1).

Fifth, the data regarding change in weight (growth rates) are limited. These outcomes could be used as a proxy for overall health as other studies have found significant differences in growth rates following incidence of disease, including bacterial arthritis.^24^ However, there was no control over the times of second (follow‐up) weighing making meaningful comparisons difficult except with a basic equation that attempts to normalise weights to a common time point. Twin data were used to tighten the variances in such observations but, even then, could not control for sex (albeit, as noted above, in the final analysis, this should have had no impact). Despite a 0.5 kg per lamb difference at the follow‐up weighing on average, in favour of NBS, the 95% CIs still overlapped so further work, with weight as a primary focus, would be needed to confirm (or refute) these observations in a statistically robust fashion.

On the other hand, the recruitment of an unparalleled number of animals for this trial (nearly 7000 lambs), with very close per‐lamb control matching, has allowed important insights into neonatal navel and ear tag protection of lambs.

First, as noted in the ‘Introduction’ section, there has not been the evidence base to prove, absolutely, that navel treatment is necessary. However, we show here that, even if iodine was no better than placebo (which is very unlikely), the more effective navel stump treatment (i.e., with NBS) saved lambs lives, and overall, therefore, navel treatment *is* necessary. However, this question can be interrogated a little further. The range of group‐level mortality, from 2.5% to 23% in all flocks, is comparable to other studies.^22^, ^25^, ^26^, ^27^, ^28^ When overall mortality was low (e.g., <5% in both groups/flock), there was no obvious suggestion that choice of navel treatment matters (Table 1) and, quite possibly, it is factors unrelated to navel (or ear tag site) treatment that are responsible for lamb deaths. Equally, however, it was apparent that, more commonly, there are risk factors that do respond to optimal navel and ear tag site treatments (Figure 1 and Table 1). The 2023/2024 lambing season was particularly badly affected by Schmallenberg virus,^29^ and, although, in the large majority of cases, Schmallenberg‐affected lambs are stillborn or euthanased immediately upon birth, and hence not eligible for recruitment in this study, it is noteworthy that some of the highest mortality overall occurred in two flocks that were affected by this virus. The 2023/2024 lambing survey of farmers^30^ indicated that it was also a particularly harsh season for weather‐related lamb losses. So, at the very least, navel treatment is a safety net when early‐day lamb survival could be negatively affected by local risk factors that may be difficult to control.

Second, as noted earlier, it is generally felt that dipping the navel is a superior method to spraying the navel, in terms of positive lamb outcomes, as dipping ensures complete coverage. This study, although not designed to test this in a matched fashion, had sufficient lamb numbers to provide an evidence base with a statistically meaningful outcome that was in favour of dipping (Table 3 and Figure 1b).

Only one farm treated the navel site by a combination of methods—with spraying and then dipping—but numbers were large (2231 lambs) allowing formal comparison to ‘gold standard’ dipping. There was no significant difference, suggesting that if spraying must be the immediate method of application, then there is value following up with dipping. More efficient, however, would be just one application by dipping initially.

Third, again as noted earlier, there is evidence that effective treatment of ear tag sites also helps to lead to favourable outcomes during lambing.^7^, ^9^, ^10^ Our findings support this notion. The inferior navel treatment (10% iodine) yielded a mortality percentage of 8.36% regardless of ear tag treatment type, but when this was restricted to 10% iodine application to both navel and ear tag sites, the mortality percentage was 9.34%. It is interesting to note that in terms of total mortality, this was just under a 3% difference compared to the NBS group, where total mortality was 6.5%. This is close to a position that, in reverse, would have required cessation of the trial for ethical reasons.

Fourth, these outcomes shine a light on precisely how navel/ear tag site treatments benefit the neonate. Figure 1 demonstrates that only the first 4 days drove the primary outcome—that is, superiority of NBS over 10% iodine in terms of mortality (11 vs. 25 deaths, respectively).

Taken together, these data strongly imply that effective prophylactic treatment of navel and ear tag sites provides protection of these ‘at risk’ areas for the first 96 hours but, thereafter, mortality causes may be unrelated. Indeed, other studies of neonatal mortality have shown that most lamb deaths occur within a week of birth, with high perinatal mortality for inside lambing associated with poor mothering or pen hygiene.^25^ Infectious disease is a key cause of neonatal lamb mortality in inside‐lambing systems,^27^ causing the death of 36% of lambs in a Norwegian study of 270 liveborn lambs that died in the first 5 days of birth, with half of these lambs being diagnosed as dying from septicaemia.^28^

Finally, this study did not attempt to address the uncommon but important issue of inappropriate antibiotic use for protection of lambs during the neonatal period. It is worth noting, however, that all neonatal lambs of one of the farms had previously received routine prophylactic antibiotic injections to prevent joint ill and that this will be discontinued in the future given that these data now show the optimal combination of navel treatment (NBS) and method (dipping) and the advantage of co‐application to ear tag sites (personal communication to F.M.L.).

## CONCLUSIONS

This well‐powered study confirms that application of an effective solution to both the navel stump and ear tag site in neonatal lambs prevents a good proportion of deaths compared with no intervention. The data also show that NBS is superior to 10% iodine in a large study that controlled for many potential confounding factors in respect of the head‐to‐head comparison. In turn, the differential data (between iodine and NBS) underscore that all of the benefit in navel and ear tag protection is within the first 96 hours of birth—thereafter, lamb deaths are likely to have other aetiologies. Post hoc analysis showed that dipping of the navel appeared more effective than spraying the navel site, consistent with prior observations.

## AUTHOR CONTRIBUTIONS

Fiona M. Lovatt designed the trial with input from all the authors. Ian S. McCrone led the ethics submission with Fiona M. Lovatt as a co‐applicant. Fiona M. Lovatt recruited all the trial sites and collected all the data, which were then shared with and reviewed independently by the authors, and the database was then locked. Ian S. McCrone developed the model for adjusted odds ratio and advised on exclusion criteria for overall data analysis. Carlos A.P. Bastos helped with data curation and analysis and generated visual representations of the results. Fiona M. Lovatt and Jonathan J. Powell wrote the manuscript. All the authors reviewed the data analysis and presentation and contributed to editing the manuscript.

## CONFLICT OF INTEREST STATEMENT

NBS technology is owned by the University of Cambridge and is licensed to NoBACZ Healthcare. J.J.P. and C.A.P.B. are inventors of the technology and may benefit financially from commercial sales of products that are based around this technology. F.M.L. is a director of Flock Health Ltd, which provides some consultancy services to NoBACZ Healthcare and may indirectly benefit from the commercial success of NBS. NoBACZ Healthcare is a spin‐out of the University of Cambridge's Department of Veterinary Medicine. J.J.P. and C.A.P.B. hold substantive and visiting University positions, respectively, as well as being employed by NoBACZ Healthcare.

## FUNDING INFORMATION

NBS was provided at no cost to trial sites by NoBACZ Healthcare. Flock Health Ltd has offered one day free‐of‐charge veterinary advice to any participating site during or after the study. Flock Health Ltd's input was re‐imbursed by NoBACZ Healthcare. NoBACZ Healthcare pays the University for use of its veterinary department site as an ‘embedded company’ but no payment has been made for this collaboration.

## ETHICS STATEMENT

The study was approved by the Ethics and Welfare Committee, Department of Veterinary Medicine, University of Cambridge (CR781).

## Supporting information



Supporting Information

Supporting Information

## Data Availability

The data that support the findings of this study are available from the corresponding author upon reasonable request.

## References

[1] West DM , Bruère AN , Ridler AL . The sheep: health, disease & production. 223. Veterinary Continuing Education; 2002.

[2] Sargison N . Sheep flock health: a planned approach. 1st ed. Wiley; 2008.

[3] Scott PR . Sheep medicine. 2nd ed. London: CRC Press; 2015.

[4] Watkins GH , Sharp MW . Bacteria Isolated from arthritic and omphalatic lesions in lambs in England and Wales. Vet J. 1998;156(3):235–238. 9883092 10.1016/s1090-0233(98)80132-9

[5] Rutherford SJ , Jeckel S , Ridler A . Characteristics of sheep flocks affected by *Streptococcus dysgalactiae* arthritis. Vet Rec. 2015;176(17):435. 10.1136/vr.102781 25724543

[6] Ridler A , Hickson R , Griffiths K , Pettigrew E , Kenyon P . Effects of *Streptococcus dysgalactiae* polyarthritis on lamb growth and mortality and risk factors for disease. Small Rumin Res. 2019;177:25–28.

[7] Smistad M , Wolff C , Tollersrud T , Tømmerberg V , Phythian C , Kampen AH , et al. Flock‐level risk factors for outbreaks of infectious arthritis in lambs, Norway 2018. Acta Vet Scand. 2020;62(1):64. 33228728 10.1186/s13028-020-00561-z PMC7686670

[8] Jackson LP , Higgins HM , Duncan JS . A cross‐sectional survey of farmer reported prevalence and farm management practices associated with neonatal infectious arthritis (“joint ill”) in lambs, on UK sheep farms. Front Vet Sci. 2024;11:1489751. 39764374 10.3389/fvets.2024.1489751 PMC11701153

[9] Veterinary Laboratories Agency . Numerous reports of metabolic disease in dairy cows. Vet Rec. 2011;168(15):401–404. 21644266 10.1136/vr.d2343

[10] Hovers K . Joint ill in lambs. Livestock. 2014;19(5):298–303.

[11] Lovatt F , Duncan J , Hinde D . Responsible use of antibiotics on sheep farms: application at farm level. In Pract. 2019;41(1):23–33.

[12] Henderson DC . Veterinary book for sheep farmers. Diamond Farm Book Publications; 2000.

[13] Hadley FB . Navel infection. In: Principles of veterinary science. W.B. Saunders Company; 1954. p. 469–471.

[14] Camp M , Renaud D , Duffield T , Gomez D , McFarlane W , Marshall J , et al. Describing and characterizing the literature regarding umbilical health in intensively raised cattle: a scoping review. Vet Sci. 2022;9:288. 35737340 10.3390/vetsci9060288 PMC9229987

[15] Robinson AL , Timms LL , Stalder KJ , Tyler HD . The effect of 4 antiseptic compounds on umbilical cord healing and infection rates in the first 24 hours in dairy calves from a commercial herd. J Dairy Sci. 2015;98(8):5726–5728. 26026760 10.3168/jds.2014-9235

[16] Fordyce AL , Timms LL , Stalder KJ , Tyler HD . The effect of novel antiseptic compounds on umbilical cord healing and incidence of infection in dairy calves. J Dairy Sci. 2018;101(6):5444–5448. 29573800 10.3168/jds.2017-13181

[17] Wieland M , Mann S , Guard CL , Nydam DV . The influence of 3 different navel dips on calf health, growth performance, and umbilical infection assessed by clinical and ultrasonographic examination. J Dairy Sci. 2017;100(1):513–524. 27865488 10.3168/jds.2016-11654

[18] Drug Enforcement Administration (DEA), Justice . Changes in the regulation of iodine crystals and chemical mixtures containing over 2.2 percent iodine. Final rule. Fed Regist. 2007;72(126):35920–35931. 17910137

[19] Moreno‐Reyes R , Feldt‐Rasmussen U , Piekiełko‐Witkowska A , da Rocha AG , Badiu C , Koehrle J , et al. The ETA–ESE statement on the European Chemicals Agency opinion on iodine as an endocrine disruptor. Eur Thyroid J. 2024;13(1):e230244. 38320401 10.1530/ETJ-23-0244 PMC10959048

[20] Sheep Veterinary Society . Supply of strong iodine (10%) for navel dressing of neonatal lambs. 2022 [cited 2024 Mar 4]. Available from: https://sheepvetsoc.org.uk/news/supply‐of‐strong‐iodine‐10‐for‐navel‐dressing‐of‐neonatal‐lambs/

[21] Simcock E . Lambing part 5 diseases of newborn lambs. NADIS livestock health bulletin . 2019 [cited 2025 Jan 16]. Available from: www.nadis.org.uk/disease‐a‐z/sheep/lambing/lambing‐part‐5‐diseases‐of‐newborn‐lambs

[22] Gascoigne E , Corbishley A , Davies P . Targeting lamb survival in commercial flocks: inspiring and effecting change. In Pract. 2023;45(1):29–36.

[23] Sargeant JM , O'Connor AM , Gardner IA , Dickson JS , Torrence ME , Dohoo IR , et al. The REFLECT Statement. Reporting guidelines for randomized controlled trials in livestock and food safety: explanation and elaboration. J Food Prot. 2010;73(3):579–603. 20202349 10.4315/0362-028x-73.3.579

[24] Lima E , Lovatt F , Green M , Roden J , Davies P , Kaler J . Sustainable lamb production: evaluation of factors affecting lamb growth using hierarchical, cross classified and multiple memberships models. Prev Vet Med. 2020;174:104822. 31751855 10.1016/j.prevetmed.2019.104822

[25] Binns SH , Cox IJ , Rizvi S , Green LE . Risk factors for lamb mortality on UK sheep farms. Prev Vet Med. 2002;52(3–4):287–303. 11849723 10.1016/s0167-5877(01)00255-0

[26] Dwyer CM . The welfare of the neonatal lamb. Small Rumin Res. 2008;76(1–2):31–41.

[27] Dwyer CM , Conington J , Corbiere F , Holmoy IH , Muri K , Nowak R , et al. Improving neonatal survival in small ruminants: science into practice. Animal. 2016;10(3):449–459. 26434788 10.1017/S1751731115001974

[28] Holmøy IH , Waage S , Granquist EG , L'Abée‐Lund TM , Ersdal C , Hektoen L , et al. Early neonatal lamb mortality: postmortem findings. Animal. 2017;11(2):295–305. 27452785 10.1017/S175173111600152X

[29] Loeb J . Gauging the impact of Schmallenberg. Vet Rec. 2024;194(4):138. 10.1002/vetr.3964 38362985

[30] Tarlinton R , Clifton R , Lovatt F . Schmallenberg virus: lambing season survey. Vet Rec. 2024;194(4):156–157. 38362982 10.1002/vetr.3980

