# Supplementary material for: Pseudorandomised controlled trial of a novel navel barrier solution versus 10% iodine to protect navel and ear tag sites of neonatal lambs
Source: Vet Rec. 2025 Dec 26;198(10):e444–52. doi: 10.1002/vetr.70169 (PMC13178406; doi:10.1002/vetr.70169)

**Supplementary Table 1:** comparison of mortality measures as estimated from absence at second weighing versus accurate records.

|  | Iodine | NBS | Total |
| --- | --- | --- | --- |
| **Mortality estimates at time of 2^nd^ weighing (Fig 1a)** | **283** (55.7%) | **225** (44.3%) | **508** (100%) |
| Accurately recorded mortality out to day 56 (Fig. 1C) | 67 (55.8%) | 53 (44.2%) | 120 (100%) |
| Accurately recorded mortality out to day 121 | 73 (57.0%) | 55 (43.0%) | 128 (100%) |

**Supplementary Table 2.** Individual flock data and associated univariate analyses.

| **Flock** | **Iodine** | |  | **NBS** | |  | **Odds Ratio** | **95% CI** | **p value** |
| --- | --- | --- | --- | --- | --- | --- | --- | --- | --- |
|  | **Total** | **Died** | **Mortality** | **Total** | **Died** | **Mortality** |  |  |  |
| **A** | 80 | 2 | 2.50% | 83 | 4 | 4.82% | 1.97 | 0.35-11.1 | 0.440 |
| **B** | 184 | 6 | 3.26% | 184 | 6 | 3.26% | 1.00 | 0.32-3.16 | 1 |
| **C** | 56 | 6 | 10.71% | 59 | 0 | 0.00% | 0.07 | 0.00-1.19 | 0.070 |
| **D** | 47 | 11 | 23.40% | 52 | 11 | 21.15% | 0.88 | 0.34-2.27 | 0.788 |
| **E** | 408 | 35 | 8.58% | 409 | 28 | 6.85% | 0.78 | 0.47-1.31 | 0.354 |
| **F** | 77 | 7 | 9.09% | 78 | 8 | 10.26% | 1.14 | 0.39-3.32 | 0.806 |
| **G** | 241 | 32 | 13.28% | 241 | 16 | 6.64% | 0.46 | 0.25-0.87 | 0.017 |
| **H** | 480 | 64 | 13.33% | 510 | 52 | 10.20% | 0.74 | 0.50-1.09 | 0.126 |
| **I** | 402 | 32 | 7.96% | 425 | 25 | 5.88% | 0.72 | 0.42-1.24 | 0.240 |
| **J** | 1116 | 77 | 6.90% | 1115 | 63 | 5.65% | 0.81 | 0.57-1.14 | 0.224 |
| **K** | 294 | 11 | 3.74% | 299 | 12 | 4.01% | 1.08 | 0.47-2.48 | 0.869 |
| **Overall** | 3385 | 283 | 8.36% | 3455 | 225 | 6.51% | 0.76 | 0.63-0.91 | 0.003 |

**Supplementary Figure 1**

Meta-analysis, treating all eleven flocks as separate studies, performed using a random effects model with inverse variance method to compare the hazard rate (HR).


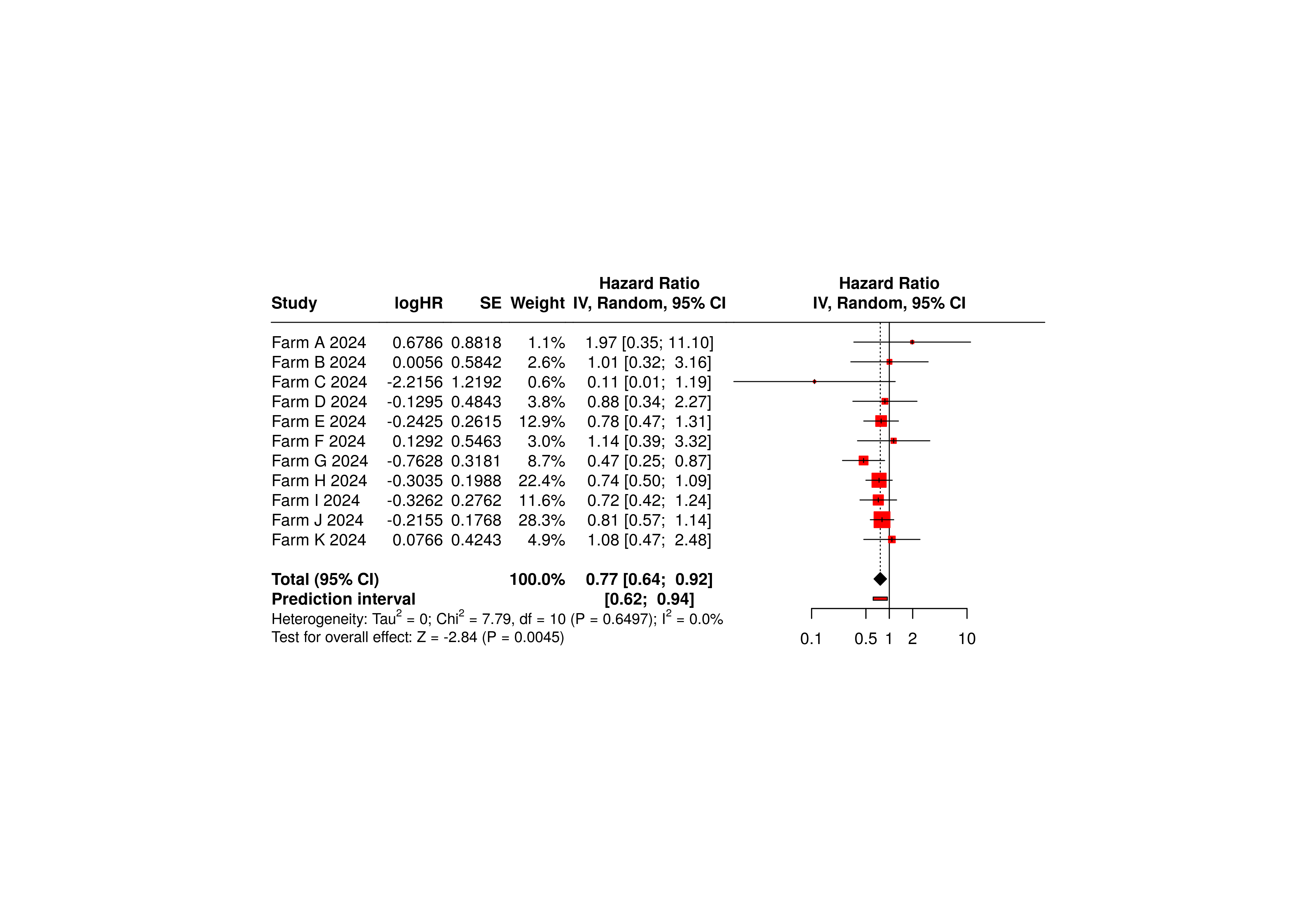

Supplement: Supplementary file 1 — Supporting Information [file VETR-198--s001.docx]
