# Supplementary material for: Pseudorandomised controlled trial of a novel navel barrier solution versus 10% iodine to protect navel and ear tag sites of neonatal lambs
Source: Vet Rec. 2025 Dec 26;198(10):e444–52. doi: 10.1002/vetr.70169 (PMC13178406; doi:10.1002/vetr.70169)

Supplementary Table 1: comparison of mortality measures as estimated from absence at second weighing versus accurate records.

	Iodine	NBS	Total
**Mortality estimates at time of 2nd weighing (Fig 1a)**	**283** (55.7%)	**225** (44.3%)	**508** (100%)
Accurately recorded mortality out to day 56 (Fig. 1C)	67 (55.8%)	53 (44.2%)	120 (100%)
Accurately recorded mortality out to day 121	73 (57.0%)	55 (43.0%)	128 (100%)

Supplementary Table 2. Individual flock data and associated univariate analyses.

	Iodine			NBS					
**Flock**	**Total**	**Died**	**Mortality**	**Total**	**Died**	**Mortality**	**Odds Ratio**	**95% CI**	**p value**
**A**	80	2	2.50%	83	4	4.82%	1.97	0.35-11.1	0.440
**B**	184	6	3.26%	184	6	3.26%	1.00	0.32-3.16	1
**C**	56	6	10.71%	59	0	0.00%	0.07	0.00-1.19	0.070
**D**	47	11	23.40%	52	11	21.15%	0.88	0.34-2.27	0.788
**E**	408	35	8.58%	409	28	6.85%	0.78	0.47-1.31	0.354
**F**	77	7	9.09%	78	8	10.26%	1.14	0.39-3.32	0.806
**G**	241	32	13.28%	241	16	6.64%	0.46	0.25-0.87	0.017
**H**	480	64	13.33%	510	52	10.20%	0.74	0.50-1.09	0.126
**I**	402	32	7.96%	425	25	5.88%	0.72	0.42-1.24	0.240
**J**	1116	77	6.90%	1115	63	5.65%	0.81	0.57-1.14	0.224
**K**	294	11	3.74%	299	12	4.01%	1.08	0.47-2.48	0.869
**Overall**	3385	283	8.36%	3455	225	6.51%	0.76	0.63-0.91	0.003

Supplementary Figure 1

Meta-analysis, treating all eleven flocks as separate studies, performed using a random effects model with inverse variance method to compare the hazard rate (HR).

Study	logHR	SE	Weight	Hazard Ratio IV, Random, 95% CI
Farm A 2024	0.6786	0.8818	1.1%	1.97 [0.35; 11.10]
Farm B 2024	0.0056	0.5842	2.6%	1.01 [0.32; 3.16]
Farm C 2024	-2.2156	1.2192	0.6%	0.11 [0.01; 1.19]
Farm D 2024	-0.1295	0.4843	3.8%	0.88 [0.34; 2.27]
Farm E 2024	-0.2425	0.2615	12.9%	0.78 [0.47; 1.31]
Farm F 2024	0.1292	0.5463	3.0%	1.14 [0.39; 3.32]
Farm G 2024	-0.7628	0.3181	8.7%	0.47 [0.25; 0.87]
Farm H 2024	-0.3035	0.1988	22.4%	0.74 [0.50; 1.09]
Farm I 2024	-0.3262	0.2762	11.6%	0.72 [0.42; 1.24]
Farm J 2024	-0.2155	0.1768	28.3%	0.81 [0.57; 1.14]
Farm K 2024	0.0766	0.4243	4.9%	1.08 [0.47; 2.48]
**Total (95% CI)**			**100.0%**	**0.77 [0.64; 0.92]**
**Prediction interval**				**[0.62; 0.94]**

Heterogeneity: $Tau^2 = 0$; $Chi^2 = 7.79$, df = 10 (P = 0.6497); $I^2 = 0.0\%$
Test for overall effect: Z = -2.84 (P = 0.0045)

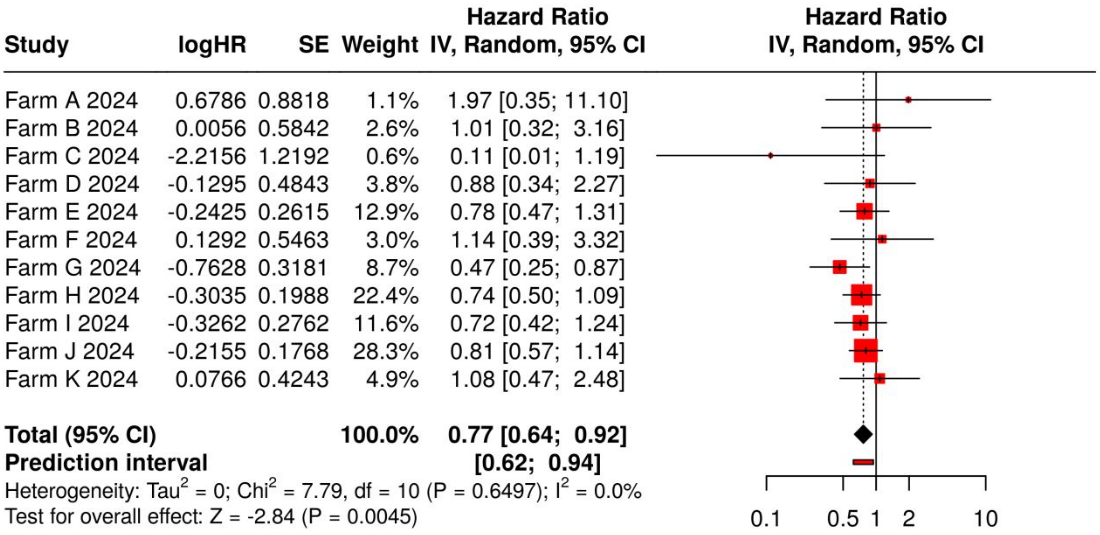

Supplement: Supplementary file 2 — Supporting Information [file VETR-198--s002.pdf]
